# Altered Cortical Synaptic Plasticity in Response to 5-Hz Repetitive Transcranial Magnetic Stimulation as a New Electrophysiological Finding in Amnestic Mild Cognitive Impairment Converting to Alzheimer’s Disease: Results from a 4-year Prospective Cohort Study

**DOI:** 10.3389/fnagi.2015.00253

**Published:** 2016-01-12

**Authors:** Alessandro Trebbastoni, Floriana Pichiorri, Fabrizia D’Antonio, Alessandra Campanelli, Emanuela Onesti, Marco Ceccanti, Carlo de Lena, Maurizio Inghilleri

**Affiliations:** ^1^Department of Neurology and Psychiatry, Sapienza University of Rome, Rome, Italy; ^2^Neuroelectrical Imaging and Brain Computer Interface Laboratory, Fondazione Santa Lucia – Istituto di Ricovero e Cura a Carattere Scientifico (IRCCS), Rome, Italy

**Keywords:** mild cognitive impairment, Alzheimer’s disease, transcranial magnetic stimulation, cortical excitability, synaptic plasticity, *N*-methyl-d-aspartate acid receptor

## Abstract

**Introduction:**

To investigate cortical excitability and synaptic plasticity in amnestic mild cognitive impairment (aMCI) using 5 Hz repetitive transcranial magnetic stimulation (5 Hz-rTMS) and to assess whether specific TMS parameters predict conversion time to Alzheimer’s disease (AD).

**Materials and methods:**

Forty aMCI patients (single- and multi-domain) and 20 healthy controls underwent, at baseline, a neuropsychological examination and 5 Hz-rTMS delivered in trains of 10 stimuli and 120% of resting motor threshold (rMT) intensity over the dominant motor area. The rMT and the ratio between amplitude of the 1st and the 10th motor-evoked potential elicited by the train (X/I-MEP ratio) were calculated as measures of cortical excitability and synaptic plasticity, respectively. Patients were followed up annually over a period of 48 months. Analysis of variance for repeated measures was used to compare TMS parameters in patients with those in controls. Spearman’s correlation was performed by considering demographic variables, aMCI subtype, neuropsychological test scores, TMS parameters, and conversion time.

**Results:**

Thirty-five aMCI subjects completed the study; 60% of these converted to AD. The baseline rMT and X/I-MEP ratio were significantly lower in patients than in controls (*p* = 0.04 and *p* = 0.01). Spearman’s analysis showed that conversion time correlated with the rMT (0.40) and X/I-MEP ratio (0.51).

**Discussion:**

aMCI patients displayed cortical hyperexcitability and altered synaptic plasticity to 5 Hz-rTMS when compared with healthy subjects. The extent of these changes correlated with conversion time. These alterations, which have previously been observed in AD, are thus present in the early stages of disease and may be considered as potential neurophysiological markers of conversion from aMCI to AD.

## Introduction

Mild cognitive impairment (MCI) may be considered as the intermediate stage between the expected cognitive decline of normal aging and the pathological decline of dementia. Several definitions of MCI have been proposed since the 1960s and many terms have been adopted in the literature to describe MCI (Kral, 1962; Reisberg et al., 1982; Flicker et al., 1991; Levy, 1994; Ebly et al., 1995). In 1999, Petersen et al. (1999) investigated MCI as a prodromal condition for Alzheimer’s disease (AD), highlighting the importance of memory complaints in incipient AD in non-demented subjects. Subsequent work enlarged the definition of MCI to include other forms of cognitive impairment that cover a range of clinical phenotypes (Winblad et al., 2004).

Mild cognitive impairment may manifest itself through a variety of symptoms. When memory loss is the predominant symptom, it is termed amnestic mild cognitive impairment (aMCI), with the single-domain (aMCIsd) subtype being used when memory is the only function affected, and the multi-domain (aMCImd) subtype when impaired memory is associated with other deficits (Petersen, 2004; Winblad et al., 2004). People with aMCI have the highest risk of conversion to AD (Bennett, 2003; Petersen, 2004; Petersen et al., 2005; Espinosa et al., 2013).

While all those who progress to AD go through a period of MCI, not all MCI leads to AD, with many MCI subjects remaining stable or returning to normality (Mitchell and Shiri-Feshki, 2009). Data on outcomes following a diagnosis of MCI suggest that the annual conversion rate to AD in clinical studies varies greatly from 10–15% (Bennett, 2003; Petersen, 2004; Petersen et al., 2005; Farias et al., 2009) to 41% (Geslani et al., 2005). The main research challenge in this field consists in identifying, from among individuals with a diagnosis of MCI, those who convert to AD, particularly those who do so more rapidly, in order to be able to treat the disease more promptly.

Several excellent studies have been designed to find the most predictive biosignature of AD pathology in MCI by investigating structural, functional, and molecular neuroimaging and cerebrospinal fluid essays of amyloid-beta (Aβ) and tau proteins (Hampel et al., 2008; van Rossum et al., 2010; Drago et al., 2011). Few works have instead studied electrophysiological biomarkers in MCI.

Transcranial magnetic stimulation (TMS) is a low-cost, non-invasive electrophysiological technique that can be used to study the mechanisms underlying cortical excitability and synaptic plasticity *in vivo* (Pascual-Leone et al., 1994; Berardelli et al., 1998; Romeo et al., 2000). It is based on the principle of electromagnetic induction of an electric field through the brain (Barker et al., 1985) that modulates the excitability of neurons (Ridding and Ziemann, 2010; Vallence and Ridding, 2014). A single TMS pulse applied over the primary motor cortex (M1) elicits a motor-evoked potential (MEP) in the contralateral target muscles (Pascual-Leone et al., 1994). The minimal intensity required to induce a MEP at rest with an amplitude of at least 50 μV with a probability of 50% defines the resting motor threshold (rMT) (Barker et al., 1985). Single TMS pulses delivered in trains are the principle of repetitive TMS (rTMS). This technique yields effects that may outlast the stimulus train and vary from inhibition to facilitation depending on the stimulus parameters adopted, and in particular on the frequency. Trains of low-frequency rTMS delivered at 1 Hz or below are believed to transiently depress synaptic efficiency, an effect that resembles that underlying long-term depression (Pascual-Leone et al., 1998). By contrast, high-frequency rTMS (hf-rTMS) (≥1 Hz) can transiently enhance cortical excitability and plasticity in a fashion resembling glutamate-dependent neurotransmission (Pascual-Leone et al., 1994, 1998; Jennum et al., 1995; Berardelli et al., 1998; Modugno et al., 2001; Kobayashi and Pascual-Leone, 2003; Inghilleri et al., 2004, 2005). When delivered in healthy subjects at 5 Hz repetitive transcranial magnetic stimulation (5 Hz-rTMS) and at a suprathreshold intensity over M1, rTMS facilitates cortical excitability, increasing the amplitude of the MEPs during the train (Berardelli et al., 1998). This facilitation outlasts the train by about 1 s, thereby providing evidence of plastic changes in the cortex. These effects seem to be related to synaptic long-term potentiation (LTP)-like mechanisms and mainly involve the glutamatergic system via the activation of postsynaptic *N*-methyl-d-aspartate acid (NMDA) receptors (NMDAr) (Kobayashi and Pascual-Leone, 2003; Inghilleri et al., 2004, 2005). This neurophysiological evidence suggests that rTMS may be a suitable tool to study alterations in synaptic plasticity and excitability within the human cortex (Hoogendam et al., 2010).

In the last two decades, several studies have adopted various TMS techniques to assess patterns of neuroplastic changes in AD, corroborating findings showing that cortical physiology is altered in AD owing to an underlying neurodegenerative process that affects cholinergic and glutamatergic neurotransmission (Di Lazzaro et al., 2002, 2004, 2006; Inghilleri et al., 2006; Nardone et al., 2006, 2008; Alberici et al., 2008; Pennisi et al., 2011). In particular, 5 Hz-rTMS has provided useful information on various aspects of altered glutamatergic neurotransmission in patients with AD (Inghilleri et al., 2006), regardless of cholinesterase inhibitor (AChEI) chronic intake (Trebbastoni et al., 2012). Consistent evidence points to decreased rMT, which reflects increased cortical excitability, and altered synaptic plasticity to rTMS as the consequences of glutamatergic system impairment in AD (Alagona et al., 2001; Ferreri et al., 2003; Di Lazzaro et al., 2004; Farlow, 2004; Inghilleri et al., 2006; Trebbastoni et al., 2012).

Dysfunctional glutamatergic neurotransmission, particularly that mediated by the NMDAr, has been studied in AD (Mota et al., 2014) though not yet in MCI. The neuropathological evidence of a striking pathology-dependent pattern of glutamatergic synaptic remodeling in both AD and MCI due to AD (Bell et al., 2007; Schaeffer and Gattaz, 2008) suggests that functional alterations in glutamatergic neurotransmission may already be detected *in vivo* in MCI. The hypothesis that these functional abnormalities may precede the development of AD and might emerge in the preclinical stages of the disease is the rationale of this research on patients with a diagnosis of MCI. The main aim of our study was, indeed, to investigate glutamatergic system functioning in MCI by evaluating cortical excitability and synaptic plasticity using 5 Hz-rTMS in subjects with a diagnosis of aMCI compared with healthy controls. Furthermore, since the data available on rTMS and other TMS techniques used as diagnostic tools in MCI (Sakuma et al., 2007; Nardone et al., 2012, 2014; Terranova et al., 2013) are scanty and contrasting, and no works have yet investigated the prognostic power of these techniques in predicting the clinical outcome in aMCI patients, the second aim of our study was to investigate any correlations between baseline responses to 5 Hz-rTMS, clinical characteristics and neuropsychological test scores and the timing of conversion to AD in a 4-year long longitudinal study.

## Materials and Methods

### Subjects

The study was conducted on 40 right-handed patients, consecutively recruited at the Department of Neurology and Psychiatry of the “Sapienza” University of Rome Umberto I Hospital, with a diagnosis of single- or multi-domain aMCI according to Petersen’s revised diagnostic criteria (Petersen, 2004; Winblad et al., 2004), and 20 healthy controls. The 12-month recruitment period ran from January to December 2007. We enrolled male or female patients with the following characteristics: age between 50 and 80 years; a self-reported history of subjective memory decline, corroborated by an informant, with gradual onset and slow progression over the previous year; an objective memory impairment as observed during the neuropsychological evaluation; a mini-mental state examination (MMSE) (Folstein et al., 1975) score ≥24; a clinical dementia rating scale (CDR) (Morris, 1993) score of 0.5 with a memory box score ≥0.5; with a geriatric depression scale (GDS) short-form (van Marwijk et al., 1995) score ≤6; with complete functional abilities of daily living as confirmed by a caregiver and measured by the activity of daily living scale (ADL) and the instrumental activity of daily living scale (IADL) (Lawton and Brody, 1969); with normal levels of serum vitamin B12, folate, and thyroid hormones; with a low cerebrovascular risk with no signs or symptoms of severe hypertension, heart disease, dyslipidemia, or diabetes; with a modified Hachinski ischemic scale (HIS) ≤4; with a magnetic resonance imaging scan (MRI) of the brain performed within the previous 6 months showing no evidence of moderate or severe chronic ischemic cerebrovascular disease rated visually on axial FLAIR images using the Fazekas scale (Fazekas et al., 1987). Only patients whose Fazekas scales were less than grade 2 were included. Healthy controls consisted of volunteers who had no neurological or psychiatric symptoms and a normal MRI of the brain. They were included if cognitive functioning was normal, as assessed by a MMSE ≥28/30 and a CDR = 0. To avoid the potential effects of any drugs on the electrophysiological parameters studied, any subjects taking drugs affecting the central nervous system, such as antidepressants, antipsychotics, anticonvulsants, AChEI, or any other dietary supplement indicated in the symptomatic treatment of MCI, were excluded.

The study was performed in compliance with the international rights of the patient and in accordance with the Declaration of Helsinki of 1990. The local ethics committee approved the experimental procedures used. All the subjects could understand and carry out the tasks required during the stimulation procedure and gave their written informed consent to participation in the study.

### Stimulation Technique

The TMS stimulation paradigm complied with the safety regulations currently recommended for TMS studies (Rossi et al., 2009). TMS was delivered through a high-frequency magnetic stimulator (Magstim Rapid – The Magstim Company Ltd., Whitland, South West Wales, UK) connected to a figure-of-eight coil placed over the motor area of the dominant hemisphere. Biphasic magnetic pulses were delivered over the primary motor area of the dominant hemisphere to find the optimal position for eliciting a MEP in the contralateral first dorsal interosseous (FDI) muscle. The coil was held tangentially to the scalp with the handle pointing back and away from the midline at 45°. The motor threshold was calculated at rest and was considered as the lowest intensity able to evoke a MEP of more than 50 μV in at least 5 out of 10 consecutive trials in the FDI muscle. Electromyographic activity was recorded through a pair of surface silver/silver chloride (Ag/AgCl) disk electrodes placed over the contralateral FDI muscle. Electromyographic signals were recorded and filtered through a Digitimer D360 amplifier (Digitimer Ltd., UK) (bandwidth 20 Hz–1 kHz) and analyzed off-line. MEPs were recorded at rest. During rTMS, participants were asked to relax the FDI muscle throughout the TMS trains. In order to ensure relaxation in the target muscle, participants were provided with visual and auditory feedback of EMG activity in the FDI. In all the subjects, rTMS was delivered in 10 trains of 10 stimuli, at a 5-Hz frequency and a stimulation intensity of 120% rMT. The inter-train interval was 2 min. The size of the MEPs evoked by rTMS was measured peak-to-peak. To quantify the percentage increase in the MEP size (MEP facilitation) during the train delivered at a 5-Hz frequency, we calculated the ratio between the amplitude of the 10th and the 1st MEP evoked by the train of stimuli (X/I-MEP ratio) (Inghilleri et al., 2006).

### Study Design

**Figure 1:**
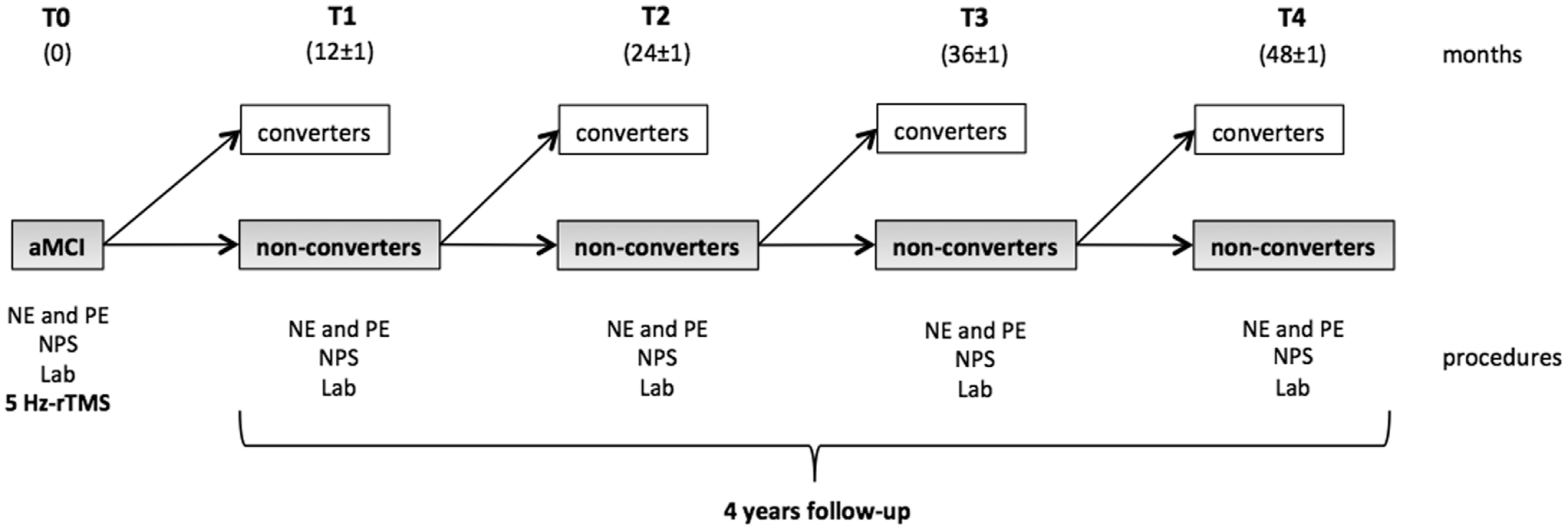
**Study design**. At the baseline (T0), all the participants enrolled underwent a neuropsychological evaluation (NPS), a physical examination (PE), blood samples (Lab), and 5 Hz-rTMS. The cohort of aMCI subjects then started the longitudinal observational period. At each time point, the patients were reassessed in order to determine whether they had converted to dementia. Subjects who fulfilled the diagnostic criteria for aMCI (non-converters) at each time point continued the study to the subsequent follow-up visit. Converters were instead excluded from the study.

Figure 1 shows the study design. All the participants underwent a physical examination, neurological assessment, and blood samples for laboratory tests at baseline (T0). Blood pressure and pulse were also measured. Subjects with aMCI also underwent a comprehensive neuropsychological evaluation (Van Gorp et al., 1986; Basso et al., 1987; Orsini et al., 1987; Spinnler and Tognoni, 1987; Carlesimo et al., 1996; Giovagnoli et al., 1996; Novelli et al., 1996; Caffarra et al., 2002, 2011; Appollonio et al., 2005), including Rey’s auditory verbal learning test (RT), the corsi block-tapping test (CT), Rey’s complex figure test (RCFT), the digit-span task (DS), the visual-search matrix test (VS), the trail-making test part A (TMT-A) and B (TMT-B), the Boston naming test (BNT); the token test (TT), verbal semantic fluency tests (VSF), verbal phonemic fluency tests (VPF), the clock drawing test (CDT), the frontal assessment battery (FAB), and Raven’s progressive colored matrices (RCPM). Patients and controls also underwent the MMSE, ADL, and IADL scales. 5 Hz-rTMS was the last experimental procedure applied at T0 in a separate session within 1 week of the clinical and neuropsychological evaluation.

The aMCI cohort was clinically followed up every 12 months (±1 month) over a period of 48 months. During the study, neither AChEI nor other substances to treat cognitive disorders were administered. At each follow-up visit (T1, T2, T3, and T4), patients underwent a clinical examination, blood testing, vital signs measurements, HIS scale, and a new neuropsychological assessment in order to assess whether conversion to AD had occurred. The clinical diagnosis of AD was made according to the National Institute of Neurologic and Communicative Disorders and Stroke-Alzheimer’s Disease and Related Disorders Association (NINCDS–ADRDA) criteria (McKhann et al., 1984). Patients who displayed steady, enhanced, or impoverished cognitive functioning but still fulfilled the criteria for MCI during re-assessments were considered as non-converters and continued in the study. Those who displayed worsened cognitive functioning with impaired autonomy levels in the activities of daily living that fulfilled the NINCDS–ADRDA criteria for a diagnosis of AD were instead included in the converters group and were consequently excluded from the study.

### Statistical Analysis

Age, rMT, amplitude of first MEP, and I/X MEP ratio in the aMCI patients and healthy controls were analyzed by means of *t*-tests for independent variables. MEP amplitudes across trains of stimuli were analyzed by means of a repeated measures analysis of variance (ANOVA), with factors “group” and “number of stimuli” as main factors. Bonferroni’s correction was applied for multiple comparisons. To investigate the value of selected neurophysiological parameters (rMT, X/I-MEP ratio) as clinical descriptors and indicators of disease progression in aMCI patients, we first performed a *t*-test for independent variables between converters and non-converters. Then, we performed a correlation analysis in converters (stratified for time of conversion during follow-up evaluation) for non-parametric data (Spearman) considering age, education, time between the onset of memory complaints and the diagnosis of aMCI, aMCI subtype, altered scores at baseline neuropsychological tests, and time of conversion to AD. Time of conversion was expressed in 12-month intervals (12, 24, 36, and 48 months).

The significance threshold was set at *p* < 0.05. Unless otherwise stated, results are expressed as means ± SD.

## Results

No side effects or adverse events related to the application of the electrophysiological stimulation procedures were reported.

Forty aMCI patients and 20 healthy controls were enrolled. Thirty-five (20 aMCIsd/15 aMCImd) of the 40 aMCI subjects completed the study, and their data were consequently included in the statistical analysis. Five patients were lost to follow-up at T1. Table 1 shows the baseline clinical, neuropsychological, and socio-demographic data of the participants.

**Table 1:** **Demographic, clinical, and neuropsychological data of patients and controls (CTR) at baseline**.

	Cognitive function explored	aMCI (Inghilleri et al., 2006)		CTR (Romeo et al., 2000)
		aMCIsd (Romeo et al., 2000)	aMCImd (Hampel et al., 2008)	
Age		74.9 ± 3.4	73.4 ± 4.0	71.3 ± 6.7
Sex (male/female)		14/6	8/7	9/11
Education (years)		8.6 ± 3.9	6.4 ± 4.5	8.0 ± 3.9
Symptoms onset (months)		28.6 ± 15.7	22.2 ± 8.5	–
MMSE		27.1 ± 1.4	26.5 ± 1.7	29.6 ± 1.3
Range (min–max)		25–28	24–29	28–30
RT learning	M	25.8 ± 2.9^a^	22.1 ± 2.8^a^	
Range (min–max)		19–28	17–27	
RT recall	M	2.7 ± 1.5^a^	1.7 ± 1.7^a^	
Range (min–max)		0–5	0–5	
CT	M, V	4.5 ± 1.5	4.25 ± 1.6	
RCFT immediate recall	M	7.9 ± 2.6	7.4 ± 2.0	
RCFT delayed recall	M	6.8 ± 2.1	6.6 ± 1.9	
DS	A	5.5 ± 2.0	5.4 ± 2.1	
VS	A	35.7 ± 3.1	33.9 ± 3.2	
TMT-A	A	55.5 ± 3.1	56.8 ± 3.0	
TMT-B	A	100.7 ± 5.2	97.6 ± 6.1	
BNT	L	34.0 ± 11.4	27.2 ± 8.1	
VPF	L	30.2 ± 9.1	20.6 ± 4.9	
VSF	L	33.4 ± 6.7	25.3 ± 8.8	
TT	L	40.2 ± 10.2	40.0 ± 12.3	
RCFT copy	V	30.2 ± 7.6	28.5 ± 6.4	
CDT	M, V	1.0 ± 0.0	1.8 ± 1.8	
FAB	F	16.8 ± 0.8	14.9 ± 1.7	
RCPM	F	42.1 ± 6.5	38.9 ± 5.5	

*Thirty-five aMCI subjects completed the longitudinal observational study. The neuropsychological battery used tests to evaluate cognitive performances in memory (M), attention (A), language (L), visuo-spatial functions (V), and frontal functions (F). The test battery included Rey’s auditory verbal learning test (RT), the corsi block-tapping test (CT), Rey’s complex figure test (RCFT), the digit-span task (DS), visual-search matrix test (VS), the trail-making test part A (TMT-A) and B (TMT-B), the Boston naming test (BNT); the token test (TT), verbal semantic fluency tests (VSF), the verbal phonemic fluency tests (VPF), the clock drawing test (CDT), the frontal assessment battery (FAB), Raven’s progressive colored matrices (RCPM), and the mini-mental state examination (MMSE). Neuropsychological test scores are expressed as means ± SD*.

*^a^Pathological scores*.

Twenty-one subjects (60%) converted to dementia over the study period. All the converters fulfilled the NINCDS–ADRDA criteria for probable AD. Table 2 shows the converters’ baseline test scores compared with those obtained at the time of conversion. When divided into the 2 subgroups, 10 (50%) of the aMCIsd and 11 (73.3%) of the aMCImd converted to AD. The mean conversion rate to AD in our sample was 15% per year when the aMCI patients were considered as a whole, and 12.5 and 18.3% in the aMCIsd and aMCImd subgroups, respectively.

**Table 2:** **Converters’ MMSE, ADL, IADL, and RT scores obtained at baseline (T0) and at the time point of conversion (conv)**.

	aMCI type	Conversion time point	MMSE		ADL		IADL		RT learning		RT recall	

			T0	Conv	T0	Conv	T0	Conv	T0	Conv	T0	Conv
1	sd	T1	25	19	6	5	5	4	27	19	4	0
2	sd	T2	27	21	6	4	5	3	25	17	5	0
3	sd	T2	25	20	6	5	5	3	28	17	2	0
4	sd	T2	27	20	6	4	5	3	22	18	1	1
5	sd	T3	25	20	6	5	8	4	26	20	4	1
6	sd	T3	27	21	6	4	5	3	19	10	0	0
7	sd	T3	28	20	6	5	5	2	28	22	4	2
8	sd	T3	28	24	6	5	5	4	28	23	2	2
9	sd	T4	27	22	6	5	5	3	28	20	4	0
10	sd	T4	29	20	6	4	5	4	28	20	2	0
11	md	T1	29	22	6	3	8	3	24	19	5	1
12	md	T1	24	19	6	3	8	3	22	15	2	0
13	md	T1	25	20	6	4	5	3	20	12	0	0
14	md	T1	25	22	6	4	5	3	27	25	0	0
15	md	T2	27	23	6	5	8	4	23	19	3	0
16	md	T2	27	20	6	4	8	6	21	17	3	0
17	md	T2	28	23	6	4	8	4	17	10	0	0
18	md	T3	27	19	6	4	5	3	20	17	0	0
19	md	T3	24	20	6	4	8	5	24	16	3	0
20	md	T4	28	22	6	5	5	3	25	16	1	0
21	md	T4	28	21	6	4	5	3	20	16	2	0

Mean			26.7	20.9					23.9	17.5	2.2	0.3
SD			1.6	1.4					3.5	3.8	1.7	0.7

*sd, single-domain; md, multi-domain; SD, standard deviation*.

No significant differences in age or in years of education were observed between patients (mean age: 74.4 ± 4.1 years; mean education: 7.5 ± 4.3) and controls (mean age: 70.7 ± 9.1 years; mean education: 8.0 ± 3.9) (*p* > 0.05). rMT was significantly lower in patients (56.8 ± 9.6) than in controls (64.1 ± 9.7) (*p* = 0.04), whereas no significant difference was observed between the two groups in the amplitude of the first MEP (patients 0.6 ± 0.6, controls 0.5 ± 0.3, *p* > 0.05). The X/I-MEP ratio was significantly lower in patients (1.2 ± 1.2) than in healthy controls (2.4 ± 1.1) (*p* = 0.01).

**Figure 2:**
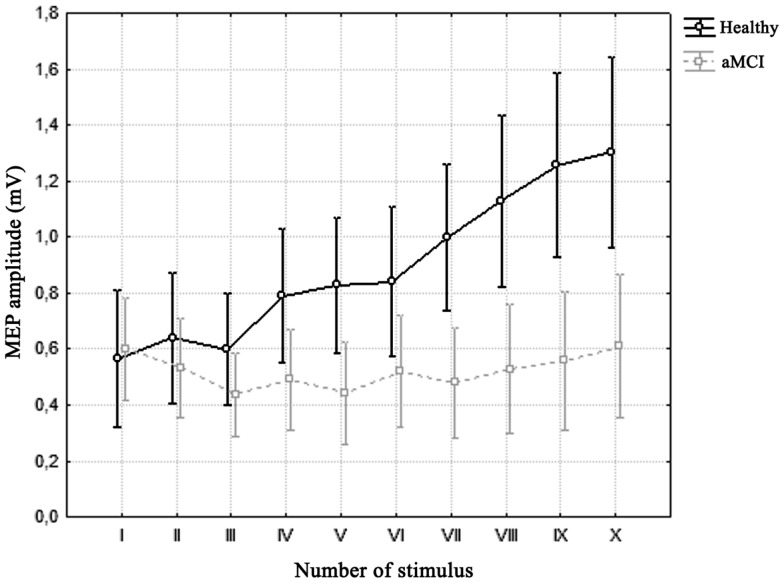
**Average motor-evoked potential (MEP) amplitude across trains of 5 Hz-rTMS in the two groups**. Vertical bars denote 0.95 confidence intervals. Repeated measures ANOVA showed a significant interaction of factors “Number of Stimulus” (*x*-axis) and “Group” (healthy vs. aMCI). Bonferroni’s correction revealed that while there was no significant difference across stimuli in the MCI group, there was a significant increase in MEP amplitude in healthy controls from the seventh stimulus onward.

Repeated measures ANOVA for MEP amplitude across 5 Hz trains revealed a significant effect of factor “group,” with a lower MEP amplitude in aMCI patients [*F*_(1, 53)_ = 5.8852, *p* = 0.01871], and a significant effect of factor “number of stimuli” [*F*_(9, 477)_ = 17.741, *p* = 0.00000]; a significant interaction of factors “group” and “number of stimuli” also emerged [*F*_(9, 477)_ = 13.115, *p* = 0.00000]. Bonferroni’s correction showed that while there was no significant change in MEP amplitude across trains in the MCI patients, a significant increase in MEP amplitude was detected in healthy controls from the seventh stimulus onward (Figure 2).

We then selected the two neurophysiological variables (rMT and X/I-MEP ratio) that showed significant differences between MCI patients and healthy controls, to investigate possible differences between converters to AD (in the clinical 4-year follow-up study) and non-converters. *T*-test for independent variables showed no significant differences (rMT converters 55.2 ± 9.3, non-converters 61.1 ± 11.1, *p* > 0.05; X/I-MEP ratio converters 1.2 ± 1.5, non-converters 1.1 ± 0.7, *p* > 0.05).

**Figure 3:**
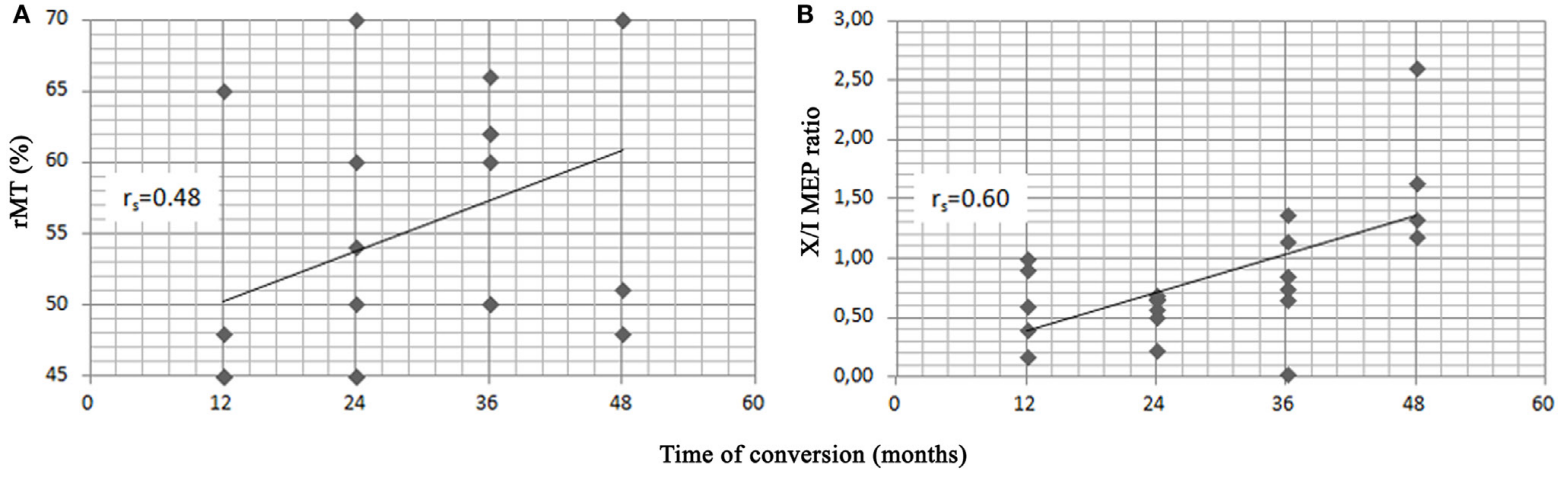
**Scatter plots of resting motor threshold (rMT) (A), X/I-MEP ratio (B) and time of conversion to AD in the 21 converters**. Spearman’s rank correlation index (*r*_s_) is shown for each variable.

Spearman’s correlation analysis was performed in converters and showed that age significantly correlated with the baseline MMSE score (−0.62), that the baseline score in the RT learning test correlated with the aMCI subtypes (−0.58), with a higher score being observed in the single-domain group, and that the time of conversion to AD correlated with MMSE score at baseline (0.43), years of education (0.44) and with the two selected neurophysiological variables, i.e., the rMT (0.48) and X/I-MEP ratio (0.60) (Figure 3). No other significant correlations were detected.

## Discussion

The application of 5 Hz-rTMS in patients with a diagnosis of aMCI revealed altered synaptic plasticity and cortical excitability, as shown by the reduction in the rMT and X/I-MEP ratio, when compared with healthy controls. Furthermore, these alterations significantly correlated with the time of conversion to AD in those patients that did convert during the follow-up.

Few previous works have investigated cortical excitability in the earlier stages of AD, including MCI, and the results of those that have are inconclusive (Sakuma et al., 2007; Nardone et al., 2008). All of these works were cross-sectional studies and adopted a range of TMS techniques on small-size samples. Our prospective study complements published data in this field by revealing a decreased rMT in aMCI patients. Similar results that have emerged from several previous studies were interpreted as the electrophysiological correlate of cortical glutamatergic dysfunctions in AD (Di Lazzaro et al., 2003a,b, 2004; Inghilleri et al., 2006). Many of these studies found that reduced rMT was a marker of increased motor cortex excitability in moderate AD (de Carvalho et al., 1997; Alagona et al., 2001; Di Lazzaro et al., 2002, 2003a, 2004, 2006; Pennisi et al., 2002; Inghilleri et al., 2006). Most authors agree that the cortical hyperexcitability described in AD is caused by the overactivation of glutamate-dependent intracortical excitatory circuits rather than by deficient intracortical inhibitory systems (Di Lazzaro et al., 2003a, 2004). Our findings showing that the rMT is decreased in aMCI suggest that a state of hyperexcitability of M1 already exists in the preclinical stages of the disease, months before conversion to AD. We speculate that altered glutamatergic functioning, which has previously been described as a potential functional compensatory mechanism in AD (Niskanen et al., 2011), may start in the early stages of the disease, and even be found in the MCI stage.

With regard to cortical plasticity within M1, we observed that the application of 5 Hz-rTMS in healthy controls induced the expected MEP facilitation, with a progressive increase in the MEP amplitude occurring during the train, whereas repetitive stimulation had no effect in subjects with aMCI, in whom the MEP amplitude remained stable throughout stimulation. The absence of 5 Hz-rTMS-induced MEP facilitation in aMCI subjects’ points to an impairment of the mechanisms underlying the induction of cortical synaptic plasticity in patients. These findings are in contrast to those previously observed in AD patients, in whom the same stimulation paradigm elicited a series of MEPs whose amplitude progressively decreased over the train of stimuli (Inghilleri et al., 2006). The altered progression of the MEPs over the train during repetitive stimulation in the aMCI group reveals an initial impairment of the mechanisms that underlie glutamate-induced synaptic potentiation. Interestingly, the extent of the alteration of these responses, as quantified by the baseline X/I-MEP ratio, correlates with the timing of conversion during follow-up, with more marked alterations in early converters. Since 5 Hz-rTMS-associated MEP facilitation resembles an experimental model of STP that is largely dependent on the activation of postsynaptic NMDAr (Castro-Alamancos and Connors, 1996; Baudry and Lynch, 2001; Inghilleri et al., 2004, 2005; Malenka and Bear, 2004), we hypothesize that the lack of MEP facilitation observed in aMCI depends on the initial impairment of NMDA-dependent cortical glutamatergic neurotransmission, which in turn leads to the inefficacy of short-term forms of enhancement in synaptic activity. Furthermore, the fact that the steady increase in MEP amplitude over the train of stimuli in aMCI patients who subsequently converted to AD differs from that previously described in the literature in AD patients (Inghilleri et al., 2006) suggests that the extent of the glutamate-dependent STP alterations we observed may be related to the stage of disease, i.e., marked in AD patients, in whom the absence of facilitation is associated with a progressive decrease in the MEP amplitude over the TMS train, and mild or incipient in MCI patients, in whom the absence of facilitation instead occurs without any significant decrease in MEP amplitude from the 1st to the 10th stimuli. The physiopathological correlates of these findings might hence depend on a specific dysfunction in NMDAr-dependent glutamatergic neurotransmission. This hypothesis is in keeping with previous studies that investigated synaptic plasticity and cortical excitability in response to TMS in healthy subjects using substances that interact directly with NMDAr (Höffken et al., 2013). Di Lazzaro et al. (2003b) demonstrated that ketamine reduces rMT, increases cortical excitability, and counteracts synaptic facilitation by progressively reducing the MEP amplitude induced by rTMS. These drug-induced responses are similar to those we observed in aMCI subjects. Since ketamine acts through the selective blockade of NMDAr, followed by an increase in non-NMDA neurotransmission, we speculate that a similar condition of imbalanced neurotransmission between the NMDA and non-NMDA systems, which favors non-NMDA activation, may occur in the brain of patients with aMCI. The fact that the amplitude of the first MEP recorded during 5 Hz-rTMS was normal in both patients and controls lends further support to a possible functional imbalance toward non-NMDA transmission, e.g., α-amino-3-hydroxy-5-methyl-4-isoxazolepropionic acid (AMPA) transmission. Indeed, the first MEP recorded mainly depends on activation of the AMPA receptor system (Di Lazzaro et al., 2003b, 2004), which appears to be spared in the preclinical stages and late stages of AD (Inghilleri et al., 2006).

Several studies supports the notion that synaptic plasticity is necessary for learning and memory (Martin et al., 2000) and activity-dependent NMDAr-associated synaptic plasticity is a prominent feature of the hippocampus (Neves et al., 2008). The hippocampus is a crucial component of the medial temporal lobe memory circuit. It is affected deeply and early in AD (Braak et al., 1998) and its degeneration leads to memory complain and prevents the acquisition of new episodic memories (Moodley and Chan, 2014). Aβ42 accumulation, the hallmark of AD pathogenesis, perturbs hippocampal LTP, decreases spine density and disrupts memory-related synapse function (Varga et al., 2015) since the early stages of AD (Hanson et al., 2015). Even though the causes that underlie NMDAr involvement in AD are largely unknown, multiple lines of evidence suggest that some of the deleterious effects of Aβ42 can be directly mediated by NMDAr widely in the brain (Scheuer et al., 1996). Aβ42 oligomers have been shown to interact with numerous regulatory proteins and directly with NMDAr, thereby causing synaptic dysfunctions (Braak et al., 1998; Neves et al., 2008; Moodley and Chan, 2014; Varga et al., 2015) and reducing glutamatergic neurotransmission (Moodley and Chan, 2014). In turn, NMDAr activation stimulates Aβ42 production (Scheuer et al., 1996; Hanson et al., 2015), which reduces the efficacy of glutamatergic transmission (Moodley and Chan, 2014) by facilitating the internalization of NMDAr (Danysz and Parsons, 2012). All these events may lead, in the early stages of the disease, to reduced functioning of NMDA receptors expressed in the synapses (Schaeffer and Gattaz, 2008; Shankar et al., 2008). In 2007, in a post-mortem immunohistochemical study, Bell and co-workers observed a paradoxical increase in glutamatergic presynaptic bouton density in persons with MCI due to AD. The authors speculated that an increase in presynaptic glutamatergic terminals indicates a type of compensatory up-regulation designed to counter the effects of pre-existing Aβ42-induced synaptotoxicity, or that the up-regulated terminals are indicative of an uncoordinated aberrant response that is not representative of concerted synaptic plasticity (Bell et al., 2007). In keeping with these hypotheses, our results in aMCI patients point to the functional inefficacy of attempts by the synapses to restore synaptic functioning and plasticity in the preclinical stages of the disease.

Another interesting finding of our research is that the altered rMT value and X/I-MEP ratio correlated with the conversion time to AD. Among aMCI patients who converted to AD, those with worse baseline TMS responses appear to be those who developed AD earlier. Higher cortical excitability and more severely impaired cortical synaptic plasticity, as demonstrated by lower rMT levels and lower X/I-MEP ratio, respectively, point to a more severe functional impairment, which in turn leads to more rapid progression to dementia. Since rMT and the X/I-MEP ratio did not correlate with one another, it may be presumed that these two TMS variables measure two independent phenomena, both of which are linked to glutamatergic system dysfunction.

The positive correlation between conversion time and baseline MMSE score and conversion time and years of education is another suggestive finding of our research. Early converters, in fact, seem to obtain lower scores at the baseline MMSE and to have a lower educational level than those that did convert later during the follow-up. These data are in line with previous works investigating neuropsychological (Arevalo-Rodriguez et al., 2015), clinical, and demographic (Tokuchi et al., 2014) predictors of conversion from MCI to AD. Interestingly, the Spearman analysis did not reveal any correlations between the rMT and the X/I-MEP ratio and the other variables studied including the MMSE score and the years of education. This finding suggests that neurophysiological parameters behave as independent variables in the prediction of early conversion to AD in aMCI.

Although further studies on larger samples are needed to confirm our preliminary data, the possibility that 5 Hz-rTMS may be used in aMCI patients to identify subjects likely to develop AD within a relatively short period of time is appealing. Patients with a diagnosis of aMCI due to AD could, by means of 5 Hz-rTMS, be classified as early or late potential converters on the basis of TMS responses. This information might prove useful not only in clinical practice but also for research purposes by offering the opportunity to conduct controlled randomized trials to evaluate the efficacy of new disease modifying therapies on the clinical progression of MCI patients with different neurophysiological phenotypes.

The strengths of this work include the high adherence rate to a strict protocol and the prospective design. The main limitation is the relatively small sample size: in our interpretation, this explains the lack of significant results in the comparison of altered TMS responses (rMT and X/I-MEP ratio) between converters and non-converters. Furthermore, as we did not adopt cerebrospinal fluid biomarkers or amyloid PET tracers in our patients, some uncertainty surrounding the accuracy of the diagnoses may arise. However, in order to reduce to a minimum possibility of including patients with preclinical cognitive impairment due to a condition other than AD, we only enrolled patients who fulfilled criteria that offer the highest predictive power of conversion from aMCI to AD in a clinical setting. Additional clinical, neuropsychological, and neuroradiological inclusion and exclusion criteria were also used to further restrict recruitment only to subjects with features often found in persons with a high risk of developing AD. The decision to use such highly specific criteria coupled with the relatively small number of participants enrolled from among the outpatients of a referral memory clinic may explain the high rate of conversion to AD in our study sample. A further limitation is the lack of follow-up neurophysiological evaluation that could provide useful information on the possible progression of the observed alterations in TMS responses.

## Conclusion

In conclusion, our data confirm that 5 Hz-rTMS is a non-invasive method that can be used *in vivo* to study changes in cortical excitability and plasticity in patients with cognitive disorders. The alterations found in patients with a diagnosis of aMCI may reflect a cortical glutamatergic system dysfunction that already exists in the prodromal stages of AD. The extent of these alterations also appears to correlate with the time of conversion to AD. One suggestive hypothesis is that a person with a clinical diagnosis of aMCI may be identified as a high or low risk subject for conversion using 5 Hz-rTMS, an electrophysiological technique that is widely available and easily applied. Further population-based studies on larger numbers of participants are needed to compare the specificity and sensitivity of 5 Hz-rTMS as a neurophysiological marker of conversion to AD in MCI with that of other pathology-specific biomarkers available for AD.

## Conflict of Interest Statement

The authors report no known conflicts of interest associated with this publication and no financial support for this work that could have influenced its outcome. We confirm that the manuscript has been read and approved by all named authors and that there are no other persons who satisfied the criteria for authorship but are not listed. We further confirm that the order of authors listed in the manuscript has been approved by all of us. We confirm that we have given due consideration to the protection of intellectual property associated with this work and that there are no impediments to publication, including the timing of publication, with respect to intellectual property. In so doing, we confirm that we have followed the regulations of our institutions concerning intellectual property.
